# The Role of Glucosinolates from Cruciferous Vegetables (Brassicaceae) in Gastrointestinal Cancers: From Prevention to Therapeutics

**DOI:** 10.3390/pharmaceutics14010190

**Published:** 2022-01-14

**Authors:** Catarina Melim, Maria R. Lauro, Isabel M. Pires, Paulo J. Oliveira, Célia Cabral

**Affiliations:** 1 Faculty of Medicine, Clinic Academic Center of Coimbra (CACC), Coimbra Institute for Clinical and Biomedical Research (iCBR), University of Coimbra, 3000-548 Coimbra, Portugal; catarina_melim@hotmail.com; 2 Center for Innovative Biomedicine and Biotechnology (CIBB), University of Coimbra, 3000-548 Coimbra, Portugal; pauloliv@ci.uc.pt; 3 Department of Pharmacy, University of Salerno, Via Giovanni Paolo II, 84084 Fisciano, Italy; lauro@unisa.it; 4 Department of Biomedical Sciences, Faculty of Health Sciences, University of Hull, Hull HU6 7RX, UK; i.pires@hull.ac.uk; 5 CNC—Center for Neuroscience and Cell Biology, University of Coimbra, 3004-517 Coimbra, Portugal; 6 Center for Functional Ecology, Department of Life Sciences, University of Coimbra, Calçada Martim de Freitas, 3000-456 Coimbra, Portugal

**Keywords:** gastrointestinal tract, gastrointestinal cancers, cruciferous vegetables, glucosinolates, sulforaphane, stability, formulation

## Abstract

The gastrointestinal (GI) tract is composed of rapidly renewing cells, which increase the likelihood of cancer. Colorectal cancer is one of the most frequently diagnosed GI cancers and currently stands in second place regarding cancer-related mortality. Unfortunately, the treatment of GI is limited, and few developments have occurred in the field over the years. With this in mind, new therapeutic strategies involving biologically active phytocompounds are being evaluated as anti-cancer agents. Vegetables such as broccoli, brussels sprouts, cabbage, cauliflower, and radish, all belonging to the Brassicaceae family, are high in dietary fibre, minerals, vitamins, carotenoids, polyphenols, and glucosinolates. The latter compound is a secondary metabolite characteristic of this family and, when biologically active, has demonstrated anti-cancer properties. This article reviews the literature regarding the potential of Cruciferous vegetables in the prevention and/or treatment of GI cancers and the relevance of appropriate compound formulations for improving the stability and bioaccessibility of the major Cruciferous compounds, with a particular focus on glucosinolates.

## 1. Introduction

### 1.1. An Overview of Gastrointestinal Cancers

**Figure 1:**
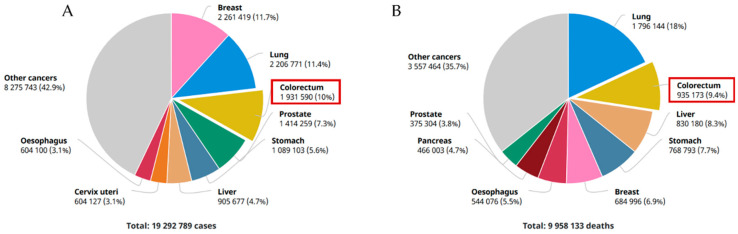
Incidence (**A**) and mortality rate (**B**) of CRC in 2020. Just under 2,000,000 people were diagnosed with CRC and around half of the cases resulted in death. Adapted from [9], with permission of the copyright holder.

Cancer continues to be a worldwide public health concern affecting millions of people each year and a leading cause of death [1]. Carcinogenesis is a multi-factorial process influenced by genetic, epigenetic, and environmental components [2]. The gastrointestinal (GI) tract is composed of a series of organs and structures responsible for several physiological functions, ranging from food digestion and absorption to protection and excretion [3]. Its surface is composed of a protective layer of rapidly renewing epithelial cells, which make GI cancers a frequently diagnosed set of tumours that affect both genders and typically develop in the stomach, liver, oesophagus, and colon/rectum [4]. Colorectal cancer (CRC) is the most common form of GI cancer, with over 800,000 estimated incident cases in women and over 1,000,000 in males in 2020, with half resulting in death (Figure 1) [5,6]. GI cancers can be hard to diagnose at early stages, impacting long-time survival, with symptoms usually appearing at later stages of the disease, culminating in a 5 year survival rate of under 20% [7,8]. The diagnosis of the disease currently involves invasive techniques, namely endoscopy, and biopsy, bringing discomfort and additional pain to the patient [8].

CRC stands as the most common type of GI cancer, the third most common cancer type, and the second leading cause of cancer-related mortality worldwide [9]. Rectal cancer alone is responsible for one third of the diagnosed cases, and a trend for an increase in the early-age (under 50 years) diagnostic of rectal cancer has been observed recently, with the age of onset dropping in prospective estimates for the USA and Europe [10]. Being mostly sporadic (60–65% of cases), a minority of the diagnosed CRC cases have a genetic component, affecting patients with a family history or those who inherited mutations that predispose and elevate the risk of developing the disease [11]. The heriditary risk for the disease is low (12 to 35%), and although ultimately unclear, the environment’s impact in the sporadic appearance of the disease is therefore of extreme relevance [11,12]. Several practices, such as alcohol intake or smoking, promote CRC development. Furthermore, known risk factors include both the lack of physical exercise and unhealthy eating habits, namely a diet mainly composed of processed foods, red meat, fat from animal sources, and low on vegetable, fruit, fibre, and calcium consumption [13,14].

Primary liver cancer is the third most frequent cause of death by cancer globally and the sixth most common type of malignancy. Hepatocellular carcinoma represents 70 to 85% of the reported cases, followed by intrahepatic cholangiocarcinoma [15,16]. The disease’s progression is aggressive and presents a poor prognosis, as the yearly fatality ratio is close to 1, meaning that most patients fail to survive a year after diagnosis, and its 5 year survival ratio is 9%, being one of the only tumours with a steady and steep incidence and mortality increase [16,17]. Furthermore, only about 5–15% of the patients are eligible for surgery, performed only in early-stage cases, not presenting cirrhosis or compromised regenerative capacity and without extrahepatic metastasis [18]. Infections with hepatitis B and C, aflatoxin B1, smoking, alcohol-related liver disease, as well as iron surplus, obesity, diabetes, and unhealthy dietary changes are linked to the origin of hepatocellular carcinomas [15,18]. As the risk factors are known, management and prevention of the cancer’s appearance are possible [19].

Currently, gastric cancer is the fifth most diagnosed malignancy, with over 1,000,000 new cases reported each year, and is the fourth leading cause of cancer-related death, primarily due to advanced-stage diagnosis [20,21]. This neoplasm is characterised by a high incidence, twice as high in males compared with the affected female population, an elevated metastasis and mortality rate, poor early diagnostic rate, and a low 5 year survival rate of less than 30% [20,22]. *Helicobacter pylori* infection is the most predominant risk factor for gastric cancer development, although in recent years, the prevalence of this bacterium has reduced amid economic development [23]. Additionally, dietary compounds such as nitrites and salt-rich foods are known contributors [24]. A high vegetable and fruit intake diet is associated with lower incidence [21,25].

Oesophageal cancer is currently the eighth most common neoplasm worldwide and the sixth in terms of worst prognosis, with its incidence increasing in the last few years [26,27]. Oesophageal squamous cell carcinoma is the most common form and is more prevalent in Asia, with oesophageal adenocarcinoma being more prevalent in Western countries and with rapidly increasing incidence [26,28]. It presents itself as an aggressive type of tumour with a low 5 year survival rate, 15–25%, and despite great advances in the treatment and diagnosis, it stands as one of the most challenging malignancies to provide care, and its prognosis is only favourable in early diagnosed cases [29,30,31]. Smoking, the consumption of alcohol, gastro-oesophageal reflux disease, unhealthy dietary habits, and obesity are risk factors [28].

Pancreatic cancer is the twelfth most common type of cancer and eighth in cancer-related deaths in 2020, as patients are typically diagnosed at advanced stages, even if the diagnosis is performed soon after symptoms appear [32]. Pancreatic cancer has the lowest survival chance out of all solid tumours, with a 5 year survival rate of just 8%, with pancreatic adenocarcinoma being the most frequent form of pancreatic cancer diagnosed, accounting for over 85% of the cases. In comparison, pancreatic endocrine tumours represent 5% of cases. Similarly to gastric and oesophageal cancers, there is an increased tendency for males to develop the disease as opposed to females [33,34]. The incidence of pancreatic cancer reaches its peak between the age of 60–80 years old, with 90% of cases being sporadic in nature. As with colorectal cancer, individuals with a family history of pancreatic cancer are at greater risk of cancer development. Smoking is the most significant environmental risk factor, but obesity, lack of exercise, and an unhealthy diet have all been linked to pancreatic cancer [35,36].

### 1.2. The Gut Microbiota and GI Cancers

The GI tract is home to a dynamic, balanced, and heterogeneous microbial ecosystem, fungi, eukaryotic viruses, and archaea, commonly named ‘gut microbiota’ or the ‘gut microbiome’ [37,38]. The great majority of this complex population is commensal or mutualistic, inhabits the intestinal tract, and is influenced by the host’s lifestyle, genetics, medication, and how the birth delivery was carried out [39]. The gut microbiota has a key role in the immune system response, digestion, and metabolization of food, production of vitamins and other relevant compounds, protection of the host against the infiltration of pathogens, regulation of neurological signalling, and the gut’s endocrine function [37]. However, an imbalance in the microbial population, also called dysbiosis, can favour the development of pathogens and disrupt host–microbiota communication, leading to several pathologies, including cancers affecting the GI tract, most notably colon cancer [40,41,42].

Several approaches can be adopted to avoid GI microbiota dysbiosis for GI cancer prevention, such as the host’s diet, pro-, pre- and postbiotics. The host’s diet significantly influences GI cancer prevention by consuming vegetables, grains, and fruits. These foods are metabolised in the gut microbiota into bioactive compounds, such as short-chain fatty acids and phytochemicals with bioactivity [37,43]. In addition, probiotics are used due to their role in maintaining microbiota homeostasis and lowering pathogenic microorganisms in the gut through immune modulation, improving the gut’s barrier function, and their anti-inflammatory/pathogenic properties [44]. Prebiotics are substrates used by the host’s microbes that produce health benefits. Poly-unsaturated fatty acids and polyphenols exhibit prebiotic potential and stimulate the growth of probiotics. Some oligosaccharides with prebiotic activity, such as plant-derived oligosaccharides (pectic- and xylo-oligosaccharides) and galactooligosaccharides, prevent pathogen colonization by inhibiting their attachment to the gut epithelial cells [45,46]. Postbiotics result from probiotics that have undergone pasteurization and, along with faecal microbiota transplantation and antibiotics, can modulate gut microbiota by protecting the intestinal epithelium, introducing a new bacterial community to the host and depleting harmful gut bacteria, respectively [37,47].

### 1.3. Current Challenges with GI Cancer Treatment and Prevention

The standard line of treatment for GI cancers involves surgery, chemotherapy, and radiotherapy [48]. Neoadjuvant chemo-radiotherapy (neo-CRT) has led to a higher survival chance in patients when compared with surgical removal of the local tumour alone in advanced stages of GI tumours. Following surgery, subsequent adjuvant therapy may be required [49]. However, neo-CRT treatment efficacy can be limited due to multidrug/therapy resistance [40]. Novel treatment strategies have been used, such as targeted therapies, immunotherapy, hormone, and antibody-based therapy [6]. Unfortunately, treatment failure remains an issue in the field, with new therapeutic strategies, including using compounds derived from phytoproducts, currently being an area of research interest. GI cancer prevention is still a largely undetermined area, as the several diseases that encompass the GI cancers differ metabolically within each other. A common prevention strategy is to implement a healthier diet and lifestyle, which maintain intestinal homeostasis by influencing the gut microbiota [1,41].

Therefore, this review aims to discuss the therapeutic and/or preventive potential behind the consumption of Cruciferous vegetables in GI cancers, with a particular focus on glucosinolates.

## 2. Brassicaceae in Cancer Prevention and Treatment

### 2.1. Brassicaceae Species

The Brassicaceae family of flowering plants, also known as ‘Cruciferae’ due to their cruciform appearance and easily recognizable conserved floral plan, consists of over 300 genera, and about 4000 species present in all continents except for Antarctica, making it one of the largest plant families available [50,51,52]. However, the diversity of the species is of uneven geographical distribution, as some relevant centres of diversity are found in the Mediterranean and the Irano-Turanian regions [53]. The Brassicaceae includes the species *Brassica oleracea*, which encompasses some popular edible vegetables such as broccoli (var. *italica*), brussels sprouts (var. *gemmifera*), cabbage (var. *capitata*), cauliflower (var. *botrytis*), and kale (var. *sabellica*). Other edible species include *Brassica rapa* (turnip), *Raphanus sativus* (radish), mustard (*Sinapis alba*), black mustard (*Brassica nigra*), and rapeseed (*Brassica napus*). In fact, the Brassicaceae family is also named the ‘mustard family’, given the pungent sulphur-based smell exuded by glucosinolate metabolites [52,53,54]. The production of these vegetables and oils is estimated to be around 70,000,000 tons per year in the European continent alone, with an average yearly consumption rate of 3–5 kg per person, and may are consumed fresh, fried, cooked, baked, or fermented [52,55]. Apart from their culinary use, traditional medicine has explored the potential of the Brassicaceae family towards the prevention and treatment of a diverse group of acute or chronic diseases, most notably cancer and metabolic syndrome [52,56].

### 2.2. Brassicaceae Bioactive Compounds

Brassicaceae vegetables are high in micronutrients, dietary fibre, phytic acid, soluble sugars, and secondary metabolites, which are biologically active phytoproducts [57,58]. Furthermore, tocopherols, carotenoids, folic acid, and vitamins C and E are common vitamins found in high quantities in cruciferous vegetables [59]. Moreover, minerals such as calcium and iron are present among the various Brassicaceae vegetables. For example, within the *Brassica oleracea* species, kale is rich in phosphorus, sulphur, chlorine, and potassium [54,57]. Table 1 describes the nutritional composition of a group of the most well-known Cruciferous vegetables.

These vegetables are rich in fibre, minerals, vitamins, and phytochemicals. When in a fresh state, these contain not only antioxidants such as vitamin C and vitamin E, but also catalase, peroxidase, and superoxide dismutase enzymes. However, the nutritional composition of Brassica vegetables is influenced by the diversity, processing, time of harvest, growth environment, and cooking conditions [61].

Polyphenols such as flavonoids, phenylpropanoids, hydroxycinnamic acids, and lignans represent a class of secondary metabolites structurally diverse, composed of at least one aromatic ring and one (or more) hydroxyl group attached to the former [54,57,58]. These polyphenolic phytoproducts have been linked with anti-cancer proprieties, as well as antioxidant activity [58]. Several anti-cancer mechanisms have been attributed to polyphenols, such as preventing oxidative stress and apoptosis induction [62]. In particular, quercetin, a flavonoid found in high concentrations in broccoli, has been found to induce mitochondrial apoptotic-dependent gastric cancer stem cell growth inhibition by blocking the PI3K-Akt signaling pathway [63]. Additionally, a population-based study in Sweden found a negative correlation between a high intake of dietary quercetin and the risk of developing non-cardia gastric adenocarcinoma. These protective effects were more prominent in females under oxidative stress, such as smoking [64].

Carotenoids and tocopherols are prevalent in Brassicaceae vegetables [65]. Carotenoids are symmetrical tetraterpenes that possess a structure composed of C40 atoms. These phytochemicals are highly pigmented (yellow, orange, red) and are also involved in the appearance of the Brassicaceae family. Their presence in these vegetables is diverse, and a wide range of these compounds’ content is observed, with lutein, lycopene, α-carotene, β-carotene, and γ-carotene presenting an antioxidant activity [55,60]. Regarding lipid-soluble tocopherols, the main compound found in Brassicaceae vegetables is α-tocopherol, but γ- and δ-tocopherols are also present [55,57]. The anti-cancer activity of these phytochemicals has been evaluated in several cancer types, including GI cancers [66]. Notably, the antioxidant β-carotene has been extensively researched for this purpose [67]. A recent study found that pre-treatment of human gastric cells with β-carotene can inhibit *Helicobacter pylori*-induced increase in cell viability, ROS production, the activity of NADPH oxidase, and TRAF1 and TRAF2 gene expression, commonly overexpressed in gastric cancer cells [68]. Epidemiologically, a case–control study performed in Korea demonstrated an inverse association between a high intake of dietary carotenoids and the risk of developing gastric cancer in females, especially regarding the consumption of dietary lycopene [69].

**Figure 2:**
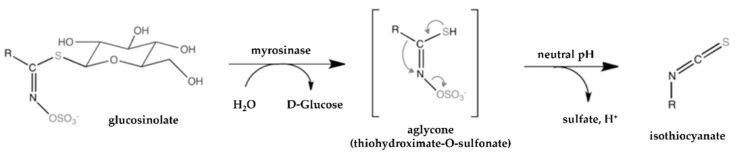
Mechanism of hydrolysis of a glucosinolate by myrosinase, originating isothiocyanates in neutral pH [71].

Glucosinolates are the most studied diverse secondary metabolites in Brassicaceae vegetables’ composition, found in high quantities in broccoli, cabbage, and brussels sprouts [61]. Glucosinolates are water-soluble anions consisting of two parts: a common β-D-thioglucose moiety and a variable aglycone side chain derived from amino acids, with the latter containing (or not) an aliphatic, aromatic, or indolyl side chain [54,70]. Plant glucosinolates are not biologically active until they are hydrolysed by myrosinase following damage to the plant (by processing techniques or chewing) releasing β-thioglucosidase, or by gut microbiota action. From these processes, some breakdown products are formed, commonly isothiocyanates (ITC), nitriles, epithionitriles, thiocyanates, and epithioalkanes, depending on the pH level and other conditions (Figure 2) [55,60].

### 2.3. Brassicaceae Phytoproducts as Anti-Cancer Therapeutics

**Figure 3:**
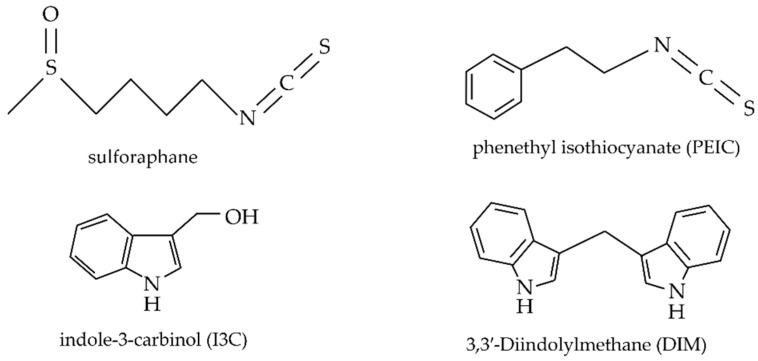
Chemical structures of the breakdown products sulforaphane, PEITC, I3C and DIM [75].

The anti-cancer properties of Brassicaceae in GI cancers and other tumour types are primarily linked to bioactive compounds following the hydrolysis of glucosinolates by myrosinase [52]. Currently, the most studied breakdown products are ITCs and indoles. ITCs are pungent phytochemicals with anticarcinogenic potential that influence the taste and smell of Brassicaceae vegetables [72,73]. Their mechanism of action includes maintaining low levels of systemic oxidative stress, inhibiting angiogenesis and cell cycle progression, and promoting apoptosis of cancerous cells [61]. In particular, sulforaphane is a potent natural ITC that presents antioxidant and anti-tumour activities [61,70]. Additionally, phenethyl isothiocyanate (PEITC) modulates the expression of several genes involved in cancer progression and development, namely genes involved in cell cycle regulation, antioxidant response, metastasis, and apoptosis [74]. Indole glucosinolates include indole-3-carbinol (I3C) and 3,3′-Diindolylmethane (DIM). I3C is a degradation product of glucobrassicin, found in numerous vegetables of the Brassica genus. Its preventive and therapeutic use has been researched against colorectal cancer and other tumor types such as prostate cancer and breast cancer (Figure 3) [53,57].

In two human cancer cell lines (HeLa and PC-3), 2-pyrrolidinone, an active compound present in *Brassica oleracea* var. *capitata*, was found to be cytotoxic in vitro and to trigger nuclear fragmentation, cell cycle arrest (G0/G1), and apoptosis, despite having reported antioxidant activity [76]. In addition, the anti-cancer effects of sulforaphane on the breast cancer cell line ZR-75-1 included growth arrest and cell cycle arrest at the G1/S transition by downregulation of CDK4. Incubation with sulforaphane led to a decrease in SERTAD1 mRNA and protein, a positive cell cycle regulator overexpressed in several cancer types [77]. Sulforaphane inhibited the cellular proliferation, migration, and cell cycle progression of the A2780 and OVCAR cell lines whilst also promoting apoptosis. In a xenograft tumour model of ovarian cancer, mice treated with sulforaphane presented smaller tumour volume and weight compared with untreated mice. Moreover, when used in combination with cisplatin, a synergistic effect was observed, with further suppression of cellular proliferation observed when compared with either treatment alone [78]. Similarly, combination therapy of sulforaphane and conventional chemotherapy agents was investigated for pancreatic cancer stem-like cells. In this regard, sulforaphane combined with cisplatin, gemcitabine, doxorubicin, or 5-flurouracil resulted in potentiating the drugs’ anti-cancer effect, with increased toxicity, apoptosis, and inhibition of the self-renewing potential of MIA-PaCa2 cells, while sparing normal cells. In vivo, combined treatment resulted in tumour growth inhibition in a pancreatic cancer stem cell xenograft. Compared with the negligible growth suppression from the single agents, this indicates that sulforaphane can sensitize the cancer stem cells to chemotherapy [79].

Erucin (ERU) is a natural isothiocyanate present in Brassica plants with H_2_S-releasing properties as well as inhibitory effects against cancer cells [80]. When used on the AsPC-1 human pancreatic adenocarcinoma cell line, the compound exhibited antiproliferative characteristics, as many H_2_S-donor agents typically do, showing cytotoxicity and preventing migration of these cells. Moreover, AsPC-1 cells are characterized by a KRAS gene mutation, leading to its hyperactivation and subsequent hyperphosphorylation and activation of the Raf/mitogen-activated protein kinase pathway and the Akt/protein kinase B pathway [81]. Interestingly, phosphorylation of ERK1/2, known to be involved in cellular proliferation and survival, was inhibited by ERU treatment [82]. Benzyl isothiocyanate (BITC) was also able to sensitize pancreatic cell lines (PANC-1 and MIAPaCa-2) to radiotherapy, with a subsequent increase in apoptosis induced by a combination of BITC and ionising radiation [83].

The therapeutic activity of 4-methylthiobutyl ITC against liver cancer has been evaluated using liver cancer cell lines HepG2, Hep3B, and Huh-7, and showed selective toxicity to the cancer cells compared with normal hepatocytes. In in vitro models, treatment with the ITC led to G2/M cell cycle growth arrest and apoptosis induction. This was observed even for chemo-resistant subpopulations of liver cancer cells, as demonstrated by the reduction in the activity of cancer stem cell marker ALDH (aldehyde dehydrogenase) and the inhibition of side population cells from the Huh-7 cell line, factors that determine drug therapy resistance. In vivo, 4-methylthiobutyl ITC oral administration was shown to be well tolerated in all the concentrations tested [84].

DIM is a nontoxic indole product from Brassica vegetables that has been investigated as an antitumor candidate for gastric cancer [85]. A study by Ye and colleagues found that DIM prevents the growth of gastric malignant in in vitro and in vivo in a dose-dependent manner. The authors also reported a novel autophagy modulation mechanism of DIM. The miR-30e, which inhibits the translation of the autophagy-related gene *ATG5*, was found to be downregulated. ATG5 is activated and forms a complex essential for the autophagy process, contributing to autophagosome formation. Thus, through the miR-30e-ATG5 autophagy modulation, gastric cancer cell proliferation is hindered [86].

It is important to note that the current literature on the anti-cancer activity of glucosinolates and other Brassica phytoproducts in GI cancers is limited. Therefore, examples of the anti-cancer activity of glucosinolates in other tumour types have been discussed, as these compounds might also have similar anti-cancer properties and mechanisms of action.

### 2.4. The Role of Brassicaceae in the Prevention of Gastrointestinal Tumours

Bioactive compounds present in vegetables and plants from the Brassicaceae family exert their preventive effects against cancer through multiple modes of action.

**Figure 4:**
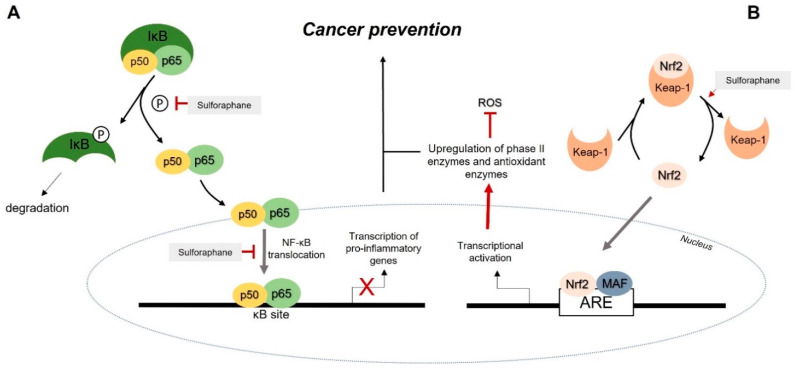
The cancer-preventive mechanism of sulforaphane through the NF-kB pathway (**A**) and the Nrf2 pathway (**B**). (**A**)—Sulforaphane can inhibit the phosphorylation of IkB and consequently the nuclear translocation of the subunit p65 of NF-κB, which affects the transcription of pro-inflammatory genes. (**B**)—Sulforaphane alters the interaction between Nrf2 and Keap-1, releasing Nrf2 and inducing its nuclear translocation. Subsequently, this transcriptional factor upregulates the expression of phase II enzymes and antioxidant enzymes. ARE: antioxidant response element; IκB: inhibitor of kappa B; Keap-1: Kelch-like ECH-associated protein 1; MAF: Nrf2 transcriptional cofactor; Nrf2: nuclear factor E2-related factor 2; ROS: reactive oxygen species. Adapted from [93,94].

One such process is the glycosylation of triterpenes, which are glycoslylated in the plant as a defence mechanism. This metabolization has anti-inflammatory and antimicrobial properties, among others [60]. ITCs inhibit NF-κB signalling, responsible for activating genes involved in inflammation, namely chemokines, cytokines such as IL-1, IL-6, IL-12, and TNF-α, and adhesion molecules [87]. One particular study revealed that sulforaphane inhibits receptor oligomerization, suppressing the TLR4 signalling cascade and leading to a downregulation of NF-κB activation. This suppression resulted in a reduction in inflammatory cytokines produced [88]. Additionally, sulforaphane and DIM repress the phosphorylation of IKK/IkB and inhibit the nuclear translocation of the subunit NF-κB p65, affecting key inflammatory mediators such as COX-2, IL-6, and TNF-α (Figure 4A) [89]. ITCs are also capable of activating the KEAP1/Nrf2 pathway, and the further activation of the ARE-mediated gene by Nrf2 can suppress the NF-κB pathway, suggesting a cross-talk mechanism between these two transcriptional factors [90].

The consumption of broccoli and its impact on non-alcoholic fatty liver disease (NAFLD) and its potential progression to hepatocellular carcinoma has also been explored. In this study, mice fed with a western diet but supplemented with freeze-dried broccoli demonstrated a lower hepatic triglyceride count and NAFLD scores, lower alanine aminotransferase plasma levels, repressed activation of hepatic CD68+ macrophages, and a decline in hepatic tumour initiation and development, when compared with mice fed with a diet lacking including broccoli [91]. Furthermore, a recent meta-analysis associated the consumption of Brassicaceae vegetables with a reduced risk of developing gastric and colorectal cancer by 19% and 8%, respectively [23].

The induction of cytoprotective enzymes, such as phase II detoxification enzymes, is a fundamental measure towards cancer prevention in the earlier development steps and may protect against DNA damage and mutagenesis [92]. As previously mentioned, phytochemicals such as sulforaphane can activate Nrf-2, which induces the expression of phase II and antioxidant enzymes such as HO-1, NQO1 and γGCS. The interaction between KEAP1/Nrf2 is altered, with sulforaphane forming thionoacyl adducts with the KEAP1 thiol content. Nrf2 is released and suffers a nuclear translocation, where it will upregulate the expression of cytoprotective enzymes, improving the antioxidant capacity while reducing oxidative stress (Figure 4B) [59,89].

Epigenetic alterations, including histone modification and DNA (de)methylation may also contribute to carcinogenesis. Several factors can mediate and modulate these alterations, including dietary derivatives such as ITCs [89]. Histone modifications, including acetylation and deacetylation, intervene in regulating the chromatin structure. Histone acetylation causes a charge change in histone proteins that allows the DNA to be easily accessible by transcriptional factors. On the other hand, if the acetyl groups are removed from histone lysine residues, access to DNA is restricted and transcription is hindered [95,96]. ITCs are competitive inhibitors of histone deacetylases (HDACs), responsible for removing the acetyl groups, thereby limiting tumorigenesis [58,96]. Additionally, alterations in DNA methylation are the most common epigenetic modifications giving rise to cancer [97]. DNA methyl transferases (DNMTs) add a methyl group near to or in the gene site. This modifies the access of the transcription machinery to the chromatin or can compromise the recruitment of methyl binding proteins, which leads to the suppression of genes, such as tumour-suppressing genes [98]. Dietary glucosinolate derivatives hinder the carcinogenic process by affecting the activity of DNMTs. For instance, sulforaphane downregulates DNMT1 and DNMT3A in breast cancer cells, repressing the expression of the human telomerase reverse transcriptase (*hTERT*) gene, which encodes the catalytic subunit of the telomerase enzyme, overexpressed in over 90% of cancers [99]. *hTERT* can be a target for amplification during the process of carcinogenesis, contributing to the dysregulation of telomerase activity and replicative immortalisation [99].

As well as the effects described above, glucosinolate bioactive product treatment has also been shown to lead to miRNA (microRNA) regulation, cell cycle arrest, and hormone receptor expression modulation as direct mechanisms of actions against tumour development [89,96]. Specifically, ITCS have the potential to modulate miRNA expression, as PEITC was found to attenuate cigarette smoking-induced altered expression of miRNAs involved in cell differentiation and proliferation, apoptosis, p53 signalling, NF-κB inhibition, and Ras activation [100]. Additionally, treatment with sulforaphane and PEITC led to G2/M arrest and decreased cell division regulators Cyclin B1, Cdc25B, and Cdc25C necessary for progression to mitosis [96].

As previously mentioned, an imbalance in the gut microbiota can lead to the development and progression of cancers, such as those affecting the GI tract. Therefore, maintaining the intestinal microbiome’s homeostasis is vital to avoid chronic inflammation, dysbiosis, and a weakened intestinal barrier, factors that can also drive carcinogenesis [2]. Through an indirect preventive manner, the flora composition of the gut microbiota may be improved by the ingestion of Cruciferous vegetables. It is known that diet dramatically influences the gut microbiota’s composition and its ability to convert glucosinolates into the bioactive metabolites [41]. The consumption of a diet including broccoli, cabbage, cauliflower, and red and green cabbage was investigated against a basal diet and found a significant difference in the gut community. Specifically, *Alistipes putredinis*, *Eggerthella* spp., *Eubacterium hallii*, and *Phascolarctobacterium faecium* were uniquely identified following the intake of cruciferous vegetables [101]. Furthermore, a previous study found that consuming broccoli decreased the *Firmicutes* relative abundance by 9% and an increase in the abundance of Bacteroidetes and Bacteroides by 10% and 8%, respectively, when compared with the control group [102]. In vivo, broccoli consumption was linked to increased activity of myrosinase in mice, which was attributed to the composition of the gut microbiota [103].

Although several studies have demonstrated the preventive potential behind the consumption of several Brassica vegetables and of their constituents towards cancer, as the promising results above show, more recent research in this matter focusing on the GI is lacking, especially concerning CRC.

## 3. Technological Strategies to Improve Stability and Bioefficacy of Cruciferous Vegetables after Oral Ingestion

When vegetables are cooked, their chemical components are quite unstable. ITC bioavailability decreases due to boiling and microwaving [104] and the amount of sulforaphane decreases by 20% after steaming, 36% after stir-frying and 88% after boiling [105]. Therefore, it is very important to choose the correct cooking preparation. Indeed, food bioactive compounds need to be bioavailable [106]. Several factors such as solubility, bioaccessibility, absorption, and transformation limit in vivo bioavailability. Furthermore, chemical transformation and the metabolism of the compounds occur in the GI tract [107]. Hence, bioavailability and bioactivity of nutraceuticals also depend on their stability in the human digestive tract. In fact, after ingestion, only the total amount or fraction of cruciferous biomass present in the gastrointestinal tract is “bioaccessibile” and available for in vivo absorption [108]. Unfortunately, chemopreventive cruciferous compounds such as polyphenols (PP), glucosinolates, and ITCs showed a significant reduction in their activity and bioavailability after oral administration [104,109,110]. Glycosidase present in the small intestine is responsible for the hydrolysis of glycoside bonds and the formation of aglycones. Furthermore, the colonic microbiome also mediates the enzymatic degradation of phenolic compounds [111]. Finally, PP undergoes non-enzymatic and enzymatic oxidation by oxides and laccases and is degraded in a pH-dependent manner [112].

As previously mentioned, when the plant tissue is cut, chopped, mixed, or chewed, a β-thioglucosidase called myrosinase is released. This enzyme can improve the bioaccessibility and bioavailability of cruciferous glucosinolates through the formation of anti-cancer metabolites such as isothiocyanates and indole-3- carbinol [113]. When this enzyme is inactivated by the high temperature of cooked vegetables or some extraction methods to formulate dietary supplements, the hydrophilic nature of glucosinolates allows it to be hydrolyzed into active metabolites by the colon intestinal microbiota [114]. Glucosinolates were also subjected to extreme conditions, including high and intensive enzymatic activities at both acidic and alkaline pH. After gastrointestinal in vitro digestion, glucosinolate levels from broccoli inflorescences, such as progoitrin, glucoraphanin, glucoalyssin, gluconapin, 4-hydroxygluco- brassicin, glucobrassicin, and 4-methoxygluco-brassicin, decreased by 69.0% in gastric medium (pH 3.0) and 12.3% after intestinal digestion (pH 7.0), due to its high degradation into nitriles and ITC products, respectively [108]. Additionally, myrosinase is an essential enzyme to release sulforaphane, the major broccoli active compound. Sulforaphane is a very unstable compound that undergoes degradation by oxygen, heat, and alkaline conditions, which makes this compound challenging to use in the nutraceutical and food industry.

In compound stability studies, PEITC from Thai Cruciferous vegetables was degraded during in vitro digestion. PEITC is a potent anti-cancer agent, effective against oesophageal, gastric, and colorectal tumors. Mechanistically, PEITC modulates phase I and phase II detoxification enzymes, but this requires it to travel intact through the liver to the target organ/tissue. At acidic pH, PEITC is degraded to phenethylamine or ITC group, which undergoes enzymatic degradation, reducing its antioxidant activity [104]. Therefore, it is fundamental to prevent the degradation of these cruciferous bioactive compounds during cooking, human digestion, and storage conditions.

There are still few studies that allow increasing the bioaccessibility and bioavailability of these products. Still, in the last ten years, the scientific community has focused its attention on increasing the bioefficiency of cruciferous plants and their compounds through micro- and nanotechnology approaches, by developing different formulations containing cruciferous compounds and extracts to produce dietary supplements and functional foods.

A study on thermal stability and improvement of bioactive efficacy was conducted by Zanoni et al. [114]. Red chicory (RCHE) and red cabbage extracts (RCAE) were obtained by extraction with different solvents, methanol or ethanol 70%. The latter extract (ethanol 70%) showed the highest thermal stability and was processed by spray-drying technology using CAPSUL^®^ modified starch as a coating material [114]. CAPSUL^®^ modified starch allowed to obtain a low-viscosity feed with high solid retention, producing a stable film around the actives [115]. Before and after encapsulation, a thermal stability test was conducted at 100 °C for 3 h in a thermostatic water bath to simulate the boiling process, then the extract powders were resuspended in 1 mL of water (pH 3.5–3.6). The results demonstrated that the encapsulation process increased the thermal stability of polyphenols and the antioxidant capacity of red chicory and red cabbage by 20–30% and 40–50%, respectively [114]. Encapsulation is also a valid technique to preserve glucosinolates and PP present in broccoli [116]. In this study, the same solvent mixture ethanol:water as before was used to produce a hybrid broccoli extract (*Brassica oleracea* var. *italica* cultivar *legacy*) rich in glucosinolates (neoglucobrassicin, glucobrassicin, 4-methoxy-glucobrassicin, and 5-methoxy-gluco- brassicin). This extract was electrosprayed in the presence of different amount of zein as wall material. Zein is a prolamine class protein of maize that functions as a polymeric amphiphile and can encapsulate various compounds, given its globular structural arrangement [117]. Both the technique and polymer were found able to achieve high thermal stability and uniform morphology when equal or less than 50% of extract was encapsulated. However, adding larger extract amounts directly affects polymer morphology and thermal stability. Pure zein or in combination with carboxymethyl chitosan was also used to obtain nanoparticles able to improve the storage stability and controlled release of two labile fat-soluble compounds from broccoli and cabbage (I3C and DIM) to food or pharmaceutical applications. This approach was chosen because it is very difficult to determine the I3C individual efficacy against cancer [118]. In fact, I3C degrades undergoing heat and light storage conditions and, after oral administration, the biological activity was partly due to its oligomerization products, such as DIM, obtained by stomach digestion [119]. The thermal stability conducted at 37 °C in a water bath confirmed this previous theory, where fast degradation rate of pure I3C compared with encapsulated zein and zein/CMCS nanoparticles was observed. A chromatography control showed that DIM resulted in major dimerization of the product after 24 h of incubation and that the pure compound is more stable than I3C under the same conditions. The results indicated that the most protection efficacy derived from chitosan/zein coating due to the presence of chitosan, which reduced the zein degradation rate during the assay. After 1 day of thermal treatment, 90% of intact I3C and only 3.26 g/mL of DIM were found in zein/chitosan coating, and 45% of intact I3C was found after 3 days. Instead, aromatic side zein protein groups and double bonds were able to absorb UV light, preserving the bioactive compounds from light degradation [118].

Sulforaphane is also unstable and sensitive to oxygen, heat, and alkaline conditions, making handling this compound difficult in the pharmaceutical industry, and reducing its success after oral administration [120,121]. To overcome these problems and improve oral efficacy against cancer, several micro or nano-encapsulation techniques and wall materials such as cyclodextrins (hydroxypropyl-β-cyclodextrin, HPCD; β-cyclodextrin, β-CD) [121,122] or maltodextrin (MD), gum arabic (GA), and carrageenan (CG) were applied [116]. Different studies showed that by complexing sulforaphane with hydroxypropyl-beta-cyclodextrin (HP), the thermal (60–90°) and chemical stability of sulforaphane also improved under acidic conditions (pH 2.0–6.0) [121,122]. Five other formulations obtained by spray-drying technique and in the presence of different wall materials (MD, GA, CG, MD/GA 25:75, and GA/β-CD 2:5; 1:10 core/wall ratio) were studied under storage conditions at 35 °C for 28 days to improve the sulforaphane stability [123]. Better stability was achieved in the presence of MD, at an inlet temperature of 170°, and a linear degradation kinetic profile [123]. Moreover, nanoencapsulation methods were applied to obtain formulations with improved in vivo sulforaphane oral bioavailability [124,125]. By applying a melt emulsification ultrasonication technique, sulforaphane-loaded nanostructured lipid carriers (SFN-NLC) were obtained. The optimized SLF-NLC revealed a higher in vitro sulforaphane release (86.52 ± 5.48%) and intestinal permeation (apparent permeability coefficient, 4.82 × 10^−4^ cm min^−1^) than active pure suspension/solution. They also showed a significant improvement of cytotoxicity (*p* < 0.05) in different cell lines, as well as antioxidant activity and oral bioavailability (5.04-fold increase) in vivo [124]. Another nanoencapsulation method based on plasma membrane vesicles obtained from cauliflower aqueous extract inflorescences was instead applied to stabilize different glucosinolates (glucoiberin, glucoraphanin, sinigrin, gluconapin, 4-hydroxy-glucobrassicin, and glucobrassicin) and other ITCs (I3C and iberin), to improve their bioaccessibility for gastrointestinal absorption [125]. In this study, a significant decrease in sulforaphane levels (from 14% to 6%) was observed in the ascending colon compared with the transversal and descending colon (*p* < 0.05). In contrast, the nanoencapsulated extract did not decrease after gastrointestinal digestion (retention of 99.4%), or colonic fermentation (*p* < 0.05), representing an important advantage for oral delivery [125]. The same biomicroencapsulation method (microencapsulation with 1 g of plasma membrane vesicles from cauliflowers) also improved the bioaccessibility of glucosinolates and sulforaphane of Bimi^®^, a new “super vegetable” Brassica hybrid variety produced by naturally crossing broccoli (*Brassica oleracea* var. *italica*) and Chinese kale (*Brassica oleracea* var. *alboglabra*) [126]. The results from the in vitro gastric digestion of the sulforaphane microencapsulated extract enriched with Bimi^®^ showed higher concentrations of the undegraded active compound found in the microencapsulated samples, suggesting that the plasma membrane vesicles from cauliflower were suitable for sulforaphane oral delivery, improving its gastrointestinal stability and bioaccessibility.

## 4. Conclusions

The gastrointestinal tract is responsible for several advanced and autonomous tasks, such as digestion, absorption, excretion, and protection. The GI lining epithelium is a continuous and rapid renewing tissue. These renewal proprieties facilitate regeneration after a lesion, but, under cancer promoting factors, such as inflammation, can be a site prone to tumour development. GI cancers such as colorectal, liver, and stomach cancers are responsible for a great majority of the cancer-related deaths every year due to high incidence, late diagnosis, and low 5 year survival rate. The standard line of treatment for GI cancers includes surgery, radiotherapy, and chemotherapy but has limitations in significantly improving patient outcomes. Therefore, further research is needed to drive a reduction in high mortality rate and improvement of patient outcomes. Several approaches that present great potential in treating GI cancers, such as various pharmacologic and biologic agents, are currently under investigation. Among them is the application of phytocompounds as preventive and/or therapeutic agents. The Brassicaceae family encompasses several edible vegetables: broccoli, cauliflower, cabbage, turnip, and radish. Chemical characterization has revealed the presence of polyphenols, carotenoids, tocopherols and vitamins. Within the secondary metabolites, glucosinolates are an important phytochemical group. Due to their anti-cancer properties, their breakdown products, isothiocyanates and indoles have been the subject of numerous research efforts. In this review, we have demonstrated examples of the anti-cancer properties of the Brassicaceae phytocompounds and summarized their preventive potential towards GI cancers. In this sense, the consumption of Brassicaceae vegetables should be promoted for their health benefits, and we hope to encourage other research groups and investigators to contribute scientific research and efforts to treat GI cancer with Brassicaceae phytoproducts. Unfortunately, their major compounds, such as glicosinolates, polyphenols, and isothiocyanates, undergo modifications in gastrointestinal conditions, which reduce their bioaccessibility, availability, and activity. Many researchers have succeeded in stabilizing these compounds using mainly micro and nanoencapsulation techniques. In the future, we expect to see chemotherapeutic agents derived from Cruciferae vegetables, appropriately formulated, to improve their anti-cancer activity and make possible their approval for clinical use, alone or in combination with already used chemotherapeutics.

## Figures and Tables

**Table 1:** Nutritional composition of *Brassica* vegetables per 100 g [57,60,61].

	Energy (Kcal)	Water Content (g)	Carbohydrates (g)	Fat (g)	Fibre (g)	Protein (g)	Minerals (mg)				Vitamins	
							Ca	Fe	K	Mg	C (mg)	Folate (µg)
**Broccoli**	34	89.3	6.6	0.37	2.6	2.8	47	0.7	316	21	89.2	63
**Brussels Sprouts**	43	86	9	0.3	3.8	3.4	42	1.4	389	23	85	61
**Cabbage**	25	92.2	5.8	0.1	2.5	1.3	40	0.5	170	12	36.6	43
**Cauliflower**	25	92	5	0.3	2	1.9	22	0.4	299	15	48.2	57
**Kale**	49	84	8.8	0.9	3.6	4.3	150	1.5	491	47	120	141
**Radish**	16	95.3	3.4	0.1	1.6	0.7	25	0.3	233	10	14.8	25
**Turnip**	28	91.9	6.4	0.1	1.8	1.2	30	0.3	191	11	21	15
